# Exercise-Induced Inguinal Hydrocele: An Unconventional Presentation of a Common Problem

**DOI:** 10.7759/cureus.13596

**Published:** 2021-02-27

**Authors:** Michael J Willcox, Brian Dahl

**Affiliations:** 1 Medicine, Tulane University School of Medicine, New Orleans, USA; 2 Medicine, Lake Erie College of Osteopathic Medicine, Erie, USA

**Keywords:** inguinal hernia, missed diagnosis, primary medical care

## Abstract

Inguinal hernias are very common. Well-established diagnostic criteria including examination and imaging are available. Ultrasound, herniography, CT, and MR imaging can provide additional diagnostic information when examination alone is not deemed sufficient. However, decision making should not be overly dependent on imaging but must factor in all relevant information. Described here is a case that would have been a missed diagnosis and an example of unconventional documentation that facilitated the patient getting their care.

## Introduction

Abdominal wall hernias are common and represent the third most frequent gastrointestinal complaint during ambulatory visits (after gastroesophageal reflux disease and constipation) [1]. Inguinal hernias are the most frequently occurring abdominal wall hernias [2] and one-third of males are diagnosed with inguinal hernias during their lifetime [3].

Examination is typically performed with the patient standing and a Valsalva maneuver is often concomitantly utilized, enhancing suspected herniations and/or producing a palpable impulse [4].

## Case presentation

A 21-year-old man developed right lower quadrant pain, nausea, and a single episode of vomiting after an otherwise uneventful jump off the back of a pickup truck. The pain and nausea spontaneously resolved with rest. In the days following, an intermittent, spontaneously resolving, right inguinal bulge was reported with associated nausea and radiating pain into the right scrotum. There was no scrotal swelling or skin changes. The patient has no prior abdominal surgeries and their medical history was unremarkable.

The patient presented a week after the initial incident to a local emergency room after repeated episodes. Hernia was initially suspected but ruled out by multiple surgeons and a radiologist after an unimpressive abdominal/genital exam and negative CT imaging with IV/enteric contrast and negative Valsalva. The patient was diagnosed with a musculoskeletal injury to the lower abdomen, prescribed conservative management, and advised to follow up with primary care if no improvement was noted.

Inguinal and scrotal sonography were performed prior to the subsequent primary care visit with no evidence of inguinal hernia and other unimpressive findings. The patient had mild increased fullness to the right inguinal region, but significant tenderness limited comprehensive examination. A repeat CT was ordered, with instructions to the patient and the radiology staff to have the patient perform 30 mins of physical activity to achieve reported changes prior to image acquisition. Patient reported difficulty achieving adequate changes at imaging and the interpretation was again unimpressive for herniation or any soft tissue bulge.

**Figure 1:**
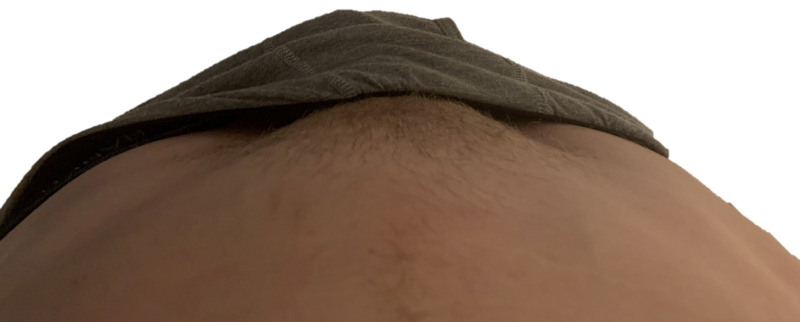
Before activity

**Figure 2:**
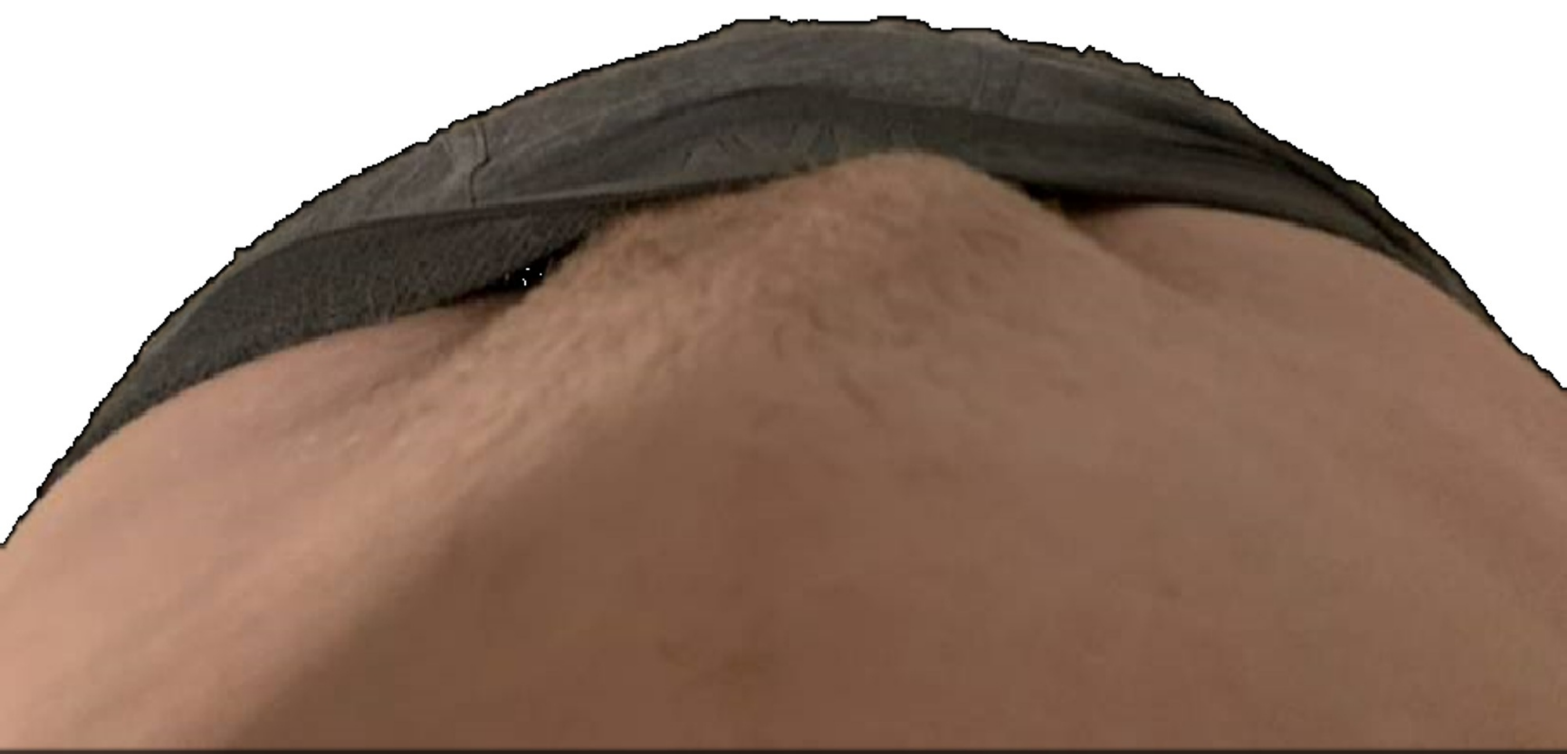
After activity Downward view of lower abdomen above lowered undergarments. No appreciable bulge noted in before view, and appreciable right inguinal bulge in after view.

The patient was instructed on specifics to acquire adequate dichotomy images of before and after aerobic activity. General Surgery was again notified with the patient provided images, which were deemed sufficient to pursue exploratory inguinotomy (Figures 1-2). Intraoperatively the patient had an indirect inguinal hernia sac and was diagnosed with a communicating inguinal hydrocele. The sack was removed and the defect repaired 12 weeks after initial presentation. The patient recovered well and returned to normal activity four weeks postoperatively with a relief of symptoms.

## Discussion

A hernia is a defect or abnormal opening in tissue. ‘-Celes’ is an ancient Greek suffix related to hernia or swelling. A hydrocele is a type of hernia where fluid collects within a pouch that produces swelling in the groin region or scrotum. Communicating hydroceles are congenital and allow fluid to freely pass with the abdomen, while noncommunicating hydroceles are often from inflammation and remain constant throughout the day. There are numerous ‘-celes’ in the abdomen/pelvis region including santorinicele, choledochocele, ureterocele, lymphocele, mucocele, rectocele, cystocele, peritoneocele, varicocele, spermatocele, hematocele, pyocele, syringocele, and hydrocele [5].

Frequently, examination alone seems to provide clear direction regarding the presence or absence of an inguinal hernia. Occult hernias can often be identified with imaging. The most used imaging modalities have the following sensitivities and specificities respectively; 86% and 77% for ultrasound, 91% and 83% for herniography, 80% and 65% for CT [6], and 94.5% and 96.3% for MRI [7]. There is a temptation when a patient has a subjective history not supported by objective examination findings and imaging to deny or minimize the severity of a defect. Providers are reminded, when there is sufficient clinical suspicion, to not fully rule out concern for inguinal hernia with imaging [8] or even specialist examination.

When examination and imaging do not support a true hernia, another consideration may be one of a “sports hernia” (athletic pubalgia, Gilmore’s groin). A misnomer, sports hernias are not a true hernia defect, but rather a strain of the muscles of the pubic aponeurosis, the lower rectus abdominus, or adductor longus [9]. However, situations like the above of a communicating hydrocele may be brought to a provider with a similar type history, and differentiation is required. This hydrocele only appeared under sustained exertion and by the time of examination and imaging, the hydrocele was no longer easily appreciable. Understanding pathophysiology will assist in differentiating the diagnosis. A communicating hydrocele like the above would be more likely to increase and decrease with time since starting or stopping exertion, versus athletic pubalgia where symptoms would be reproduced with isometric stress, for example, forced adduction against examiner’s resistance [4]. 

Also, seeking unconventional documentation, in this case, specific instructions for dichotomous images acquired by the patient outside of the controlled healthcare setting, may assist in getting a patient their needed care.

## Conclusions

While consultations with specialists and diagnostic imaging is very helpful to rule in/out conditions, if sufficient suspicion exists, physicians should be persistent in their diagnostic workup pursuits and utilize unconventional means if necessary. If the patient reports a condition that is insufficiently presented during medical examinations, the patient should be encouraged to acquire photographic or video proof to aid in the workup and communication with specialists.
